# Understanding the spatial distribution of trichiasis and its association with trachomatous inflammation—follicular

**DOI:** 10.1186/s12879-019-3935-1

**Published:** 2019-04-30

**Authors:** Rebecca Mann Flueckiger, Emanuele Giorgi, Jorge Cano, Mariamo Abdala, Olga Nelson Amiel, Gilbert Baayenda, Ana Bakhtiari, Wilfrid Batcho, Kamal Hashim Bennawi, Michael Dejene, Balgesa Elkheir Elshafie, Aba Ange Elvis, Missamou François, André Goepogui, Khumbo Kalua, Biruck Kebede, Genet Kiflu, Michael P. Masika, Marilia Massangaie, Caleb Mpyet, Jean Ndjemba, Jeremiah M. Ngondi, Nicholas Olobio, Patrick Turyaguma, Rebecca Willis, Souleymane Yeo, Anthony W. Solomon, Rachel L. Pullan

**Affiliations:** 1 London School of Hygiene and Tropical Medicine, London, UK; 2 Lancaster Medical School, Lancaster University, Lancaster, Lancashire UK; 3 Ophthalmology Department, Ministry of Health, Maputo, Mozambique; 4 Trachoma Program, Ministry of Health, Kampala, Uganda; 5 Task Force for Global Health, Decatur, GA USA; 6 Programme National de Lutte contre les Maladies Transmissibles, Ministère de la Santé, Cotonou, Benin; 7 Prevention of Blindness Program, Federal Ministry of Health, Khartoum, Sudan; 8 Michael Dejene Public Health Consultancy Services, Addis Ababa, Ethiopia; 9 National Program for Prevention of Blindness, Federal Ministry of Health, Khartoum, Sudan; 10 Programme National de la Santé Oculaire et de la lutte contre l’Onchocercose, Abidjan, Côte d’Ivoire; 11 Direction de Lutte contre la Maladie, Kinshasa, Ministere de la Santé Publique, Kinshasa, Democratic Republic of Congo; 12 Programmes National de Lutte contre l’Onchocercoses et les autres Maladies Tropicales Négligées, Ministère de la Sante, Conakry, Guinea; 13 Blantyre Institute for Community Outreach, Blantyre, Malawi; 14 Federal Ministry of Health, Addis Ababa, Ethiopia; 15 Ministry of Health, Lilongwe, Malawi; 16 Sightsavers Nigeria, Kaduna, Nigeria; 17 Department of Ophthalmology, Jos University, Jos, Nigeria; 18 RTI International, Dar es Salaam, United Republic of Tanzania; 19 Nigeria Trachoma Elimination Program, Federal Ministry of Health, Abuja, Nigeria

**Keywords:** Trachoma, Trichiasis, Blindness, Visual impairment, Neglected tropical disease, Epidemiology, Global trachoma mapping project

## Abstract

**Background:**

Whilst previous work has identified clustering of the active trachoma sign “trachomatous inflammation—follicular” (TF), there is limited understanding of the spatial structure of trachomatous trichiasis (TT), the rarer, end-stage, blinding form of disease. Here we use community-level TF prevalence, information on access to water and sanitation, and large-scale environmental and socio-economic indicators to model the spatial variation in community-level TT prevalence in Benin, Cote d’Ivoire, DRC, Guinea, Ethiopia, Malawi, Mozambique, Nigeria, Sudan and Uganda.

**Methods:**

We fit binomial mixed models, with community-level random effects, separately for each country. In countries where spatial correlation was detected through a semi-variogram diagnostic check we then fitted a geostatistical model to the TT prevalence data including TF prevalence as an explanatory variable.

**Results:**

The estimated regression relationship between community-level TF and TT was significant in eight countries. We estimate that a 10% increase in community-level TF prevalence leads to an increase in the odds for TT ranging from 20 to 86% when accounting for additional covariates.

**Conclusion:**

We find evidence of an association between TF and TT in some parts of Africa. However, our results also suggest the presence of additional, country-specific, spatial risk factors which modulate the variation in TT risk.

## Background

Trachoma is a blinding disease caused by recurrent ocular *Chlamydia trachomatis* infection, an organism that produces chronic inflammation of the tarsal conjunctiva. This is characterised by sub-epithelial follicles, which may meet the definition for the sign trachomatous inflammation—follicular (TF) [1]. TF is the sign whose prevalence in 1–9-year-olds is used to determine whether public health-level interventions against active (inflammatory) trachoma are needed [2]. Through repeated reinfection [3, 4], conjunctival scarring may develop, eventually causing the eyelashes to turn inward and touch the globe, a state known as trachomatous trichiasis (TT). In-turned eyelashes that abrade the cornea can result in corneal opacity and blindness [1]. Corrective surgery [5, 6] or epilation [7] are used to manage TT.

Ocular chlamydial transmission is declining in many countries [8–11] suggesting exceptional progress in interrupting the transmission cycle. Until recently TT prevalence was only evaluated within the context of TF surveys [12]. As TT plays an essential role in trachoma elimination, it remains important to focus on areas where TT is still a public health problem, even in the absence of TF.

The pathogenesis of trachoma, implicitly conceptualized within WHO recommendations for district-level interventions, is of repeated episodes of active trachoma incrementally increasing the cumulative risk of TT. It should be noted that this may be a simplistic outlook on the complicated pathway to TT and additional elements may influence progression. However, active trachoma is a pre-requisite on TT’s causal pathway, with moderate to high prevalences of TF being a proxy for current transmission of ocular *C. trachomatis*, and TT a proxy for historic transmission. The prevalence levels of these signs are therefore signals for *C. trachomatis* transmission intensity at different times (TF is current, and TT is historic and cumulative). Even though TF prevalence and TT prevalence are markers of transmission at different time points or over different time scales, in areas where antibiotic mass drug administration (MDA) for trachoma [13] has not yet occurred, it is often assumed that ocular *C. trachomatis* transmission intensity has remained more or less constant over decades, and that TF prevalence and TT prevalence will therefore closely correlate. This assumption is reasonable if access to water, sanitation, hygiene and anti-chlamydial antibiotics at community level have been constant or have changed only gradually. However, such an assumption is not always valid [14, 15].

Many national programmes [8] have successfully reduced TF prevalence in children aged 1–9 years below the elimination threshold of 5% [16] in some or many districts. To eliminate trachoma as a public health problem, district-level TT prevalence must also be reduced below 0.2% in adults aged ≥15 years [16]. Whether or not active trachoma and TT are public health problems are two separate questions. In Nigeria, for example, 94 local government areas in six states mapped through the Global Trachoma Mapping Project (GTMP) yielded district-level TF prevalence estimates below the elimination threshold and district-level TT prevalence estimates above the threshold [17–23]. This is attributable to historic transmission intensity being considerably higher than the contemporary one. It is important to better understand the factors associated with high TT burden, so as to develop more targeted control interventions. Understanding where TT cases are likely to occur could help to guide strategic placement of TT intervention services.

Thanks to the GTMP, there has been an increasing availability of high quality geolocated trachoma and water, sanitation and hygiene (WASH) data. The GTMP was launched in December 2012 with the aim of mapping the global prevalence of trachoma in all suspected-endemic districts, through completion of population based prevalence surveys. It systematically collected trachoma and WASH data across 1546 districts in 29 countries, nearly exclusively in areas where control activities, including antibiotic MDA, had not yet occurred [24]. These data can be used to further our understanding of TT distribution.

In this study, we attempted to identify risk factors that, in addition to TF, might associate with variation in community-level TT prevalence. To this end we fit binomial mixed models, with random effects at community level, to GTMP baseline data from ten countries. We then test for residual spatial correlation and, in countries where this is detected, use geostatistical methods in order to model the variation in TT prevalence between countries.

## Methods

### Data

Ten GTMP collaborating countries provided data for this study: Benin, Cote d’ Ivoire, Democratic Republic of the Congo (DRC), Ethiopia, Guinea, Malawi, Mozambique, Nigeria, Sudan and Uganda. Data provided were from 15,051 clusters (or communities) within 624 trachoma elimination intervention-naïve evaluation units (EUs) (Table 1). Individual-level information on the presence or absence of TF and TT, as well as water and sanitation access of geolocated households, were provided.

**Table 1:** Summary of GTMP data included in the analysis

Country	No. of communities	TF in children aged 1–9 years			TT in adults aged ≥15 years		
		No. examined	No. positive (%)	No. communities prevalence ≥5%	No. examined	No. positive (%)	No. communities with prevalence ≥0.2%
Benin	213	18,781	1594 (8.5%)	94 (44.1%)	16,170	254 (1.6%)	89 (41.8%)
Cote d’Ivoire	256	17,658	1829 (10.4%)	174 (68%)	18,771	39 (0.2%)	26 (10.2%)
DRC	1023	74,142	7022 (9.5%)	610 (59.6%)	52,200	1137 (2.2%)	511 (50%)
Ethiopia	4480	186,308	40,131 (21.5%)	3119 (69.6%)	289,230	4711 (1.6%)	2154 (48.1%)
Guinea	295	19,488	832 (4.3%)	98 (33.2%)	21,955	66 (0.3%)	53 (18%)
Malawi	1948	82,185	3437 (4.2%)	561 (28.8%)	110,815	358 (0.3%)	259 (13.3%)
Mozambique	696	34,602	2133 (6.2%)	297 (42.7%)	35,895	155 (0.4%)	117 (16.8%)
Nigeria	5364	337,962	10,070 (3%)	1105 (20.6%)	371,928	4815 (1.3%)	2035 (37.9%)
Sudan	667	33,830	1394 (4.1%)	172 (25.8%)	40,501	327 (0.8%)	197 (29.5%)
Uganda	109	6019	183 (3%)	26 (23.9%)	7445	21 (0.3%)	20 (18.3%)
Total	15,051	810,975	68,625 (8.5%)	6256 (41.6%)	964,910	11,883 (1.2%)	5461 (36.3%)

Community-level TT prevalence was calculated as the ratio between the number of adults aged ≥15 years with trichiasis in at least one eye and the number of adults aged ≥15 years examined. Community-level TF prevalence was calculated as the ratio between the number of children aged 1–9 years with TF in at least one eye and the number of children aged 1–9 years examined.

**Fig. 1:**
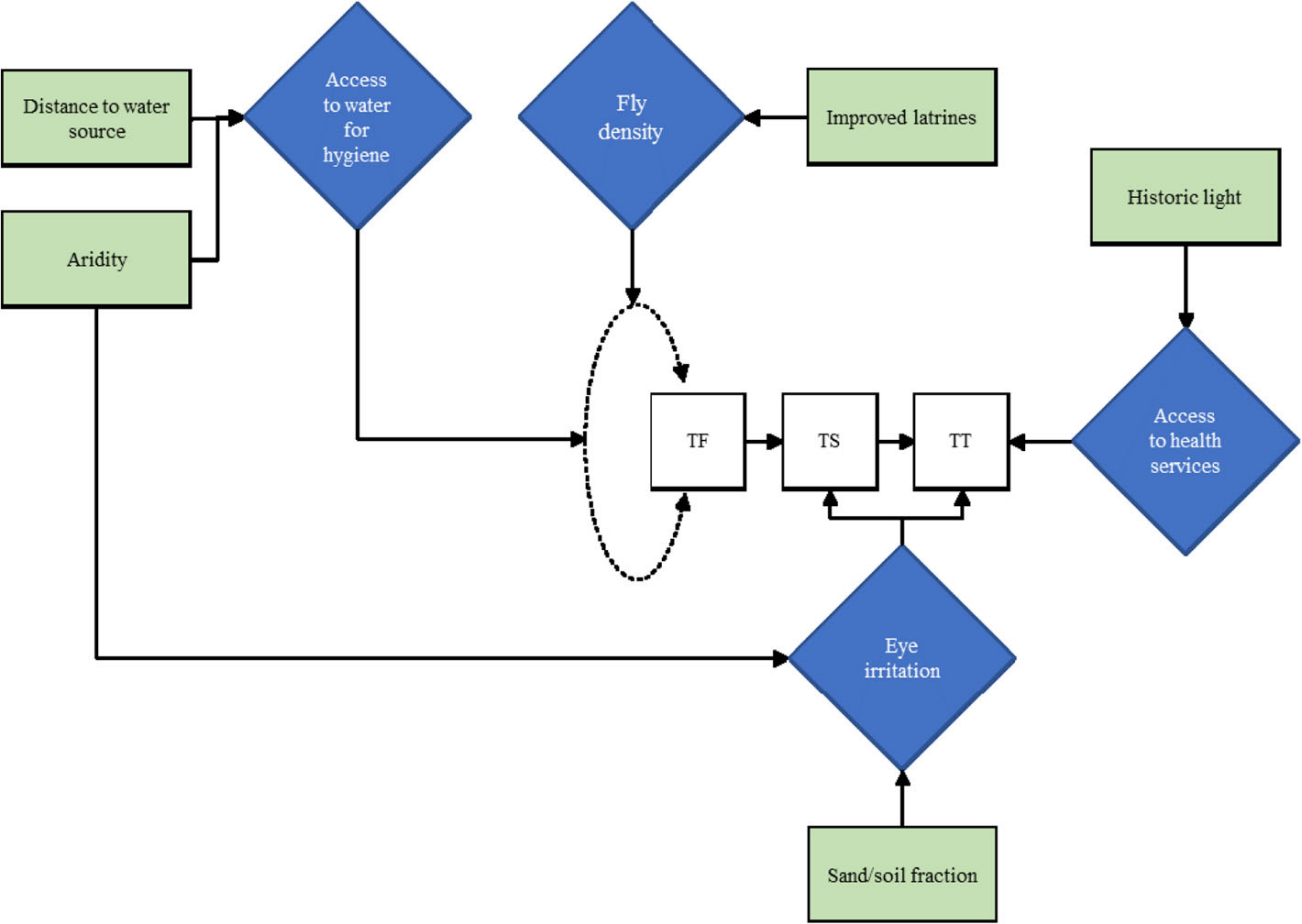
Conceptual framework of environmental risk factors influencing progression to TT

Physical and social environmental factors are hypothesized to play an important role in the natural history of trachoma. These factors could conceivably alter rate of progression to TT (Fig. 1).

Facial cleanliness is a well-established association of TF [25–30]. Access to water is necessary to facilitate personal hygiene practices. Previous studies have found an association between distance to water and risk of trachoma [25, 31–33]. There is mixed evidence on higher density populations of *Musca sorbens,* the fly vector for ocular *C. trachomatis*, being associated with a greater risk of trachoma [27, 34]. *M. sorbens* prefers to breed on human faeces left exposed on the soil [35, 36] and so it may be that latrine ownership has a protective association against active trachoma [37]. For this analysis, community-level WASH indicators were created from the GTMP household-level WASH dataset (Appendix 1). The categorization of these indicators was informed by the WHO/UNICEF Joint Monitoring Programme for Water Supply and Sanitation (JMP) [38]. We calculated the prevalence of access to each categorized WASH indicator.

Previous studies have shown that lower precipitation levels and higher temperatures can lead to an increase in the risk of TF [39]. Therefore, we selected climate-related factors, including annual total precipitation, mean temperature, aridity index and potential evapo-transpiration for this analysis. Gridded maps at 1 km^2^ resolution of annual total precipitation and mean temperature were extracted from the WorldClim database [40]. The aridity index and potential evapo-transpiration (PET) raster datasets of 1 km^2^ resolution, were obtained from the Consortium for Spatial Information (CGIAR-CSI) [41]. CGIAR-CSI modelled aridity index and PET using the data available from WorldClim as input parameters.

It has been suggested that frequent sandstorms that occur in some areas of Sudan cause eye trauma [42]. Irritation of the eyes leads to rubbing with fingers which could potentially accelerate the progression of TT. Hence, in our analysis we consider the proportion of sand in topsoil as a potential risk factor for TT. These data were obtained from the ISRIC-World Soil Information project included in the Harmonized Soil Map of the World [43].

We speculate that access to healthcare and other services are associated with developed infrastructure, and therefore sought an infrastructure indicator. Light density at night has been shown to be correlated with local economic activity and gross production rate at different scales [44, 45]. Night light emission captured by the Operational Linescan System instrument on board a satellite of the Defence Meteorological Satellite Program was used as a proxy measure of poverty across Africa [46, 47]. A gridded map of straight line distances to stable lights, namely night light emissivity > 0, was subsequently produced from the raw night light raster for 1997. This historic year was chosen because we were interested in a measure of infrastructure during the childhood of survey participants, rather than that at the time of the surveys themselves and the mean age of participants is 36 years.

All the aforementioned environmental datasets were derived from georeferenced raster files, and converted to a standardized resolution of 5 km × 5 km. The georeferenced data were linked in ArcGIS 10.1 (ESRI, Redlands, CA, USA). When shrinkage of spatial resolution was needed for the 1 km^2^ resolution covariates, we estimated the mean value in a 5 km × 5 km window using the aggregate tool in the Spatial Analyst toolbox of ArcGIS 10.1 (Appendix 2).

To identify collinearity among the selected variables, we used the variance inflation factor (VIF) [48], defined as

$$ {VIF}_j\kern0.5em =\kern0.5em \frac{1}{1-{R}_j^2} $$

where R_j_^2^ is the fraction of explained variance in the j-th explanatory variables by the other explanatory variables.

### Model formulation

Let *p*_*i*_ denote the probability of having TT, *β*_0_ is the intercept and *U*_*i*_ is community-level unstructured random effects (let i denote the i-th community). We fit the following nested binomial mixed models, where γTFi is the regression coefficient for the effect of TF prevalence on the log-odds of TT:

$$ M1:\mathit{\log}\ \left(\frac{p_i}{1-{p}_i}\right)={\beta}_0+{U}_i; $$

$$ M2:\log \left(\frac{p_i}{1-{p}_i}\right)={\beta}_0+\upgamma {TF}_i+{U}_i; $$

$$ M3:\log \left(\frac{p_i}{1-{p}_i}\right)={\beta}_0+\upgamma {TF}_i+\sum \limits_{j=1}{\beta}_j{d}_{ij}+{U}_i, $$

where *d*_*ij*_ in *M3* are the explanatory variables described in the previous section. We use the log-likelihood ratio test to select among the three models defined above.

In fitting M3, we also carried out variable selection using a backward stepwise approach, starting from the mixed effects model with all the variables included. The likelihood-ratio test was used to test for the significance of each variable, with terms removed one by one until all those remaining were significant at 5% level.

To assess the presence of residual spatial correlation, we first obtained a point estimate of the community-level unstructured random effects *U*_*i*_ from the best model identified in the previous step, and then computed the empirical semi-variogram. A semi-variogram provides insights into the rate of decay of spatial autocorrelation in the data. It does this by computing the mean squared difference between pairs of residuals as a function of the distance between their associated geographical locations. A flat semi-variogram is interpreted as evidence against the presence of spatial correlation. To test for spatial correlation more formally, we also generated 95% confidence intervals under the assumption of spatial independence. These intervals were obtained by computing semi-variograms on 1000 randomly permuted point estimates of *U*_*i*_, while holding the geographical locations fixed.

In cases where we found evidence of spatial correlation, we fitted geostatistical binomial logistic models, in which *U*_*i*_ was modelled as a spatial Gaussian process with a stationary and isotropic correlation function. All the geostatistical models were fitted in the PrevMap [49] package.

## Results

The output for the cluster-level tests suggested that temperature, precipitation, aridity index, and PET interact with one another (Table 2). Since aridity was highly correlated with each of these indicators, we retained this variable and excluded the remainder.

**Table 2:** Multicollinearity test results for gridded covariates

Variable	VIF
Annual mean temperature	5.4
Annual total precipitation	47.6
Aridity Index	62.3
Potential evapo-transpiration	6.4
Euclidean distance to ground water	1.2
Sand/soil fraction	2.0
Stable Night Light (1997)	1.2
Accessibility	1.3

The strength of association for variables in the full mixed effects model varied between countries (Table 3). There was very strong evidence of association (*P* < 0.05) between community-level TF prevalence and TT prevalence in all countries except Guinea and Uganda. In contrast, there was evidence of association with access to latrines in 4 of 10 countries (DRC, Ethiopia, Mozambique and Nigeria (*p* < 0.01)), with access to improved latrines in 2 countries (Nigeria and Uganda (*p* < 0.05)), and with water source variables in 3 countries (Ethiopia, Nigeria and Guinea (p < 0.05)). Observed relationships with environmental factors were equally heterogeneous, with associations observed with aridity index in DRC, Ethiopia, Nigeria and Sudan; and with sand/soil fraction only in Benin and Ethiopia. Night light was associated in DRC, Ethiopia, Mozambique and Nigeria.

**Table 3:** Relative increase in odds derived from a multivariate binomial logistic model where community-level prevalence of TT in adults aged ≥15-years is dependent on a 10% increase in community-level prevalence of TF in children aged 1–9 years

Covariate	Benin		Cote d’Ivoire		DRC		Ethiopia		Guinea		Malawi		Mozambique		Nigeria		Sudan		Uganda	
	OR	*p*-value	OR	p-value	OR	p-value	OR	p-value	OR	p-value	OR	p-value	OR	p-value	OR	p-value	OR	p-value	OR	*p*-value
TF prevalence	1.779	< 0.001	1.854	0.010	1.527	< 0.001	1.226	< 0.001	1.427	0.311	1.486	0.001	1.739	< 0.001	1.196	< 0.001	1.638	< 0.001	2.157	0.163
Reported use of latrines for defecation by household adults	0.596	0.087	0.909	0.421	0.902	< 0.001	1.046	< 0.001	1.067	0.254	0.835	0.034	1.082	0.001	1.051	< 0.001	0.983	0.410	1.104	0.470
Improved latrines	1.664	0.109	1.153	0.324	0.961	0.110	0.990	0.489	0.950	0.362	0.830	0.116	0.973	0.412	0.976	< 0.001	0.998	0.963	1.259	0.020
Improved water source	0.978	0.554	0.925	0.280	0.978	0.067	1.015	0.005	1.022	0.683	1.061	0.111	1.019	0.285	1.011	0.078	1.008	0.644	0.979	0.883
Improved water source on property	1.328	0.191	0.854	0.469	0.948	0.758	1.011	0.801	1.075	0.708	1.026	0.942	0.852	0.244	0.944	< 0.001	0.870	0.151	0.000	0.055
Water source on property	0.805	0.283	1.522	0.052	0.951	0.618	1.031	0.506	1.083	0.699	0.916	0.792	1.135	0.283	1.023	0.023	1.052	0.628	812.073	0.058
Water source distance more than 30 min	0.948	0.105	1.033	0.688	1.015	0.224	1.008	0.170	1.133	0.031	0.992	0.844	1.015	0.446	0.993	0.406	0.981	0.318	1.283	0.263
Aridity Index	0.854	0.402	0.904	0.729	0.919	0.006	0.881	< 0.001	0.903	0.121	0.978	0.779	0.902	0.070	0.565	< 0.001	2.293	< 0.001	2.095	0.400
Sand/soil fraction	1.962	0.033	1.444	0.450	1.002	0.977	0.798	< 0.001	0.973	0.899	1.112	0.526	1.047	0.551	0.977	0.438	0.950	0.457	0.540	0.226
Stable night light (1997)	0.293	0.200	10.257	0.371	1.311	0.003	0.807	0.003	0.397	0.414	1.538	0.640	2.160	0.001	2.121	< 0.001	1.295	0.265	16.041	0.259

Community-level household prevalence of improved sanitation and hygiene facilities as well as gridded covariates were included along with community-level prevalence of TF

The nested mixed effects models (Table 4) show that when TF was added as a fixed effect, the proportional reduction in variance ranged from 0.06 (Nigeria) to 0.42 (Benin). When environmental risk factors were added, the proportional change in variance ranged from 0.25 (Ethiopia) to 0.79 (Cote d’Ivoire). In all countries, variance continued to decrease as TF and then environmental risk factors were added to the model.

**Table 4:** Comparison of variance explained by each mixed effects model

			Null model	TF only model	TF prevalence + risk factors model
Benin		Variance	2.25	1.3	1.09
	Proportional reduction	Null model		0.42	0.52
		TF only model			0.16
Cote d’Ivoire		Variance	13.75	10.28	2.92
	Proportional reduction	Null model			0.79
		TF only model			
DRC		Variance	1.52	1.23	1.05
	Proportional reduction	Null model		0.19	0.31
		TF only model			0.15
Ethiopia		Variance	1.22	1.01	0.92
	Proportional reduction	Null model		0.17	0.25
		TF only model			0.09
Guinea		Variance	1.88	1.87*	1.37
	Proportional reduction	Null model			0.27
		TF only model			
Malawi		Variance	2.17	1.96	1.59
	Proportional reduction	Null model		0.1	0.27
		TF only model			0.19
Mozambique		Variance	4.45	3.52	3.06
	Proportional reduction	Null model		0.21	0.31
		TF only model			0.13
Nigeria		Variance	1.93	1.82	0.99
	Proportional reduction	Null model		0.06	0.49
		TF only model			0.46
Sudan		Variance	1.98	1.85	1.15
	Proportional reduction	Null model		0.07	0.42
		TF only model			0.38

The best models, selected using the likelihood ratio test, are shown in Table 5. DRC, Ethiopia and Nigeria maintained the largest number of variables significant at the 5% level. These three countries also had the largest quantities of data available.

**Table 5:** Relative increase in odds derived from a multivariate binomial logistic model where community-level prevalence of TT in adults aged ≥15-years and older is dependent on a 10% increase in community-level prevalence of TF in children aged 1–9 years. Community-level household prevalence of improved sanitation and hygiene facilities as well as gridded covariates were included along with community-level TF

Covariate type	Covariates	Benin		Cote d’Ivoire		DRC		Ethiopia		Guinea	
		OR	*p*-value	OR	*p*-value	OR	*p*-value	OR	*p*-value	OR	*p*-value
*C. trachomatis* transmission	TF prevalence	1.834	< 0.001	1.417	0.187	1.566	< 0.001	1.227	< 0.001		
WASH	Latrine defecation					0.895	< 0.001	1.045	< 0.001		
	Improved latrines										
	Improved water source							1.014	0.005		
	Improved water source on property										
	Water source on property										
	Water source distance more than 30 min									1.102	0.048
Large scale environmental	Aridity Index					0.911	0.001	0.879	< 0.001		
	Sand/soil fraction							0.795	< 0.001		
Poverty	Stable night light (1997)					1.409	< 0.001	0.806	0.003		
Covariate type	Covariates	Malawi		Mozambique		Nigeria		Sudan		Uganda	
		OR	p-value	OR	p-value	OR	p-value	OR	p-value	OR	p-value
*C. trachomatis* transmission	TF prevalence	1.481	< 0.001	1.721	< 0.001	1.193	< 0.001	1.640	< 0.001		
WASH	Latrine defecation	0.819	< 0.001	1.085	< 0.001	1.056	< 0.001				
	Improved latrines					0.977	< 0.001			1.251	0.006
	Improved water source										
	Improved water source on property					0.955	0.001				
	Water source on property					1.018	0.039				
	Water source distance more than 30 min										
Large scale environmental	Aridity Index					0.570	< 0.001	2.752	< 0.001		
	Sand/soil fraction										
Poverty	Stable night light (1997)			2.155	0.001	2.093	< 0.001				

**Fig. 2:**
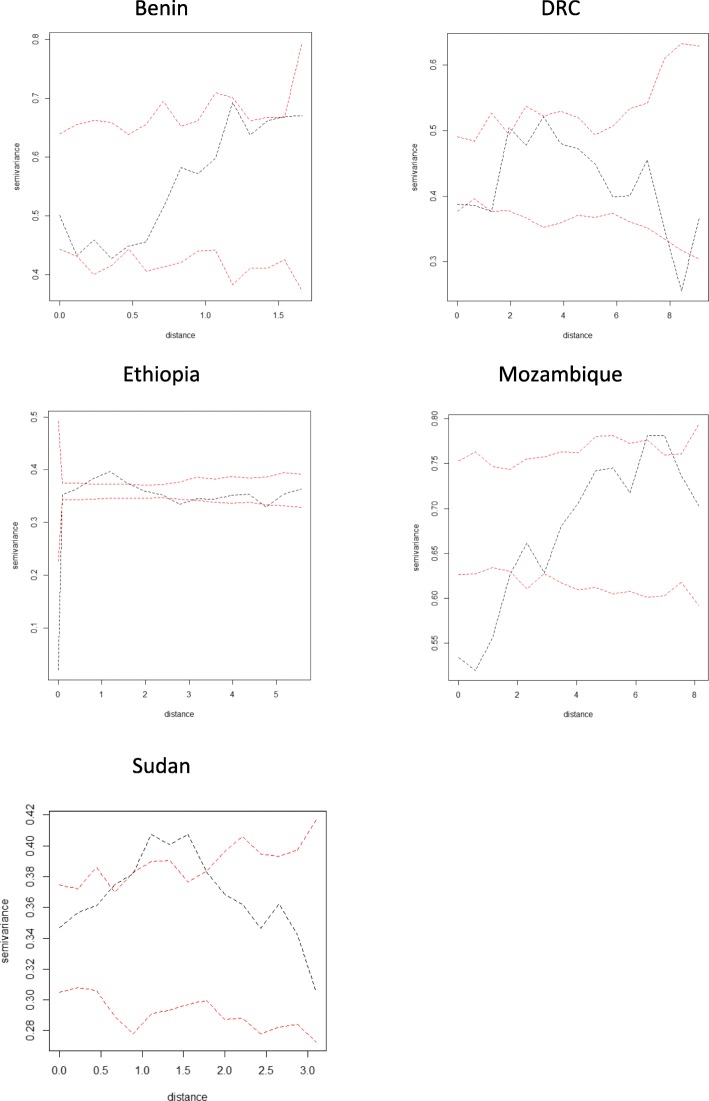
Semi-variograms were generated with Pearson’s residuals derived from the best fitting non-spatial mixed methods model. The 95% confidence intervals (red dashed lines) and semi-variogram (black dashed line) created through generating 1000 simulations are displayed here. All distances are in kilometres

Semi-variograms generated with Pearson’s residuals of the best fitting non-spatial binomial models suggest presence of residual spatial correlation in Benin, DRC, Ethiopia, Mozambique and Sudan. The 95% confidence intervals generated under the assumption of spatial independence demonstrate spatial correlation in these countries (Fig. 2).

The distance at which spatial correlation fell below 5% ranged from 3.0 km (in Ethiopia) (95% credible interval 1.6–6.0 km) to 14.2 km (in Mozambique) (95% credible interval 3.3–76.4 km), corresponding with a very rapid decline in spatial correlation with distance at larger scales, after accounting for covariates (Table 6).

**Table 6:** Scale of community-level TT prevalence spatial correlation in kilometres when accounting for covariates significant at the 5% level, by country with 95% confidence intervals

Country	scale of spatial correlation	95% confidence intervals
Benin	3.2 km	0.8–12.8 km
DRC	7.7 km	3.0–20.1 km
Ethiopia	3.0 km	1.6–6.0 km
Mozambique	14.2 km	3.4–48.9 km
Sudan	2.8 km	0.9–10.8 km

## Discussion

We investigated factors associated with community-level TT prevalence after considering the community-level TF prevalence and spatial dependency, to try and understand what causes this variation. We demonstrated considerable variation in the relationship between community-level TF and TT. When accounting for other covariates, the mixed effects models demonstrated a strong association between community-level TF and TT in eight of ten countries. These models estimate that a 10% increase in community-level TF prevalence is associated with an increase in the odds for TT of 20 to 86%, varying with setting. Benin, Cote d’Ivoire and Mozambique had exceptionally high increments in odds ratios with increasing TF, whereby a 10% increase in community-level TF prevalence was associated with an increase in TT odds of 78, 86 and 74% respectively. These high increments in odds ratios for TT lead us to speculate that reductions in TF prevalence in these environments will be quickly followed by a reduction in the incidence of TT. The relatively low increments in odds ratios in Ethiopia and Nigeria, where a 10% increase in community-level TF prevalence associated with an increase in TT odds of only 23 and 20% respectively, suggest a slight disconnect between historic and current transmission. This could be a signal of (1) a change in transmission dynamics over time, (2) population movement, (3) a pointer to the fact that we are using all-trichiasis as the dependent variable, rather than only trichiasis due to trachoma or (4) other factors are influencing TT aside from *C. trachomatis*. Further analysis is needed to explore the influence on the relationships that we observed here of including data on the presence or absence of trachomatous conjunctival scarring in eyes with trichiasis.

Importantly, in these models, the proportion of variance explained by TF ranges from 6% (in Nigeria) to 42% (in Benin). This range highlights the complex relationship of the distribution of TT and the distribution of TF. Environmental covariates, on average, explain an additional 9% (in Ethiopia) to 46% (in Nigeria) of variance. Our models suggest that while community-level TF prevalence is generally the strongest single predictor of TT, it does not fully explain the variation in community-level TT prevalence, and implying that occasionally, high-TT-prevalence populations will be found where TF is rare. It has been widely observed that dry conditions (parameterized in our analysis as a low aridity index) is a risk factor for TF in children [31, 50–52]. We found an extension of this association in three of our countries, in the form of an association of low aridity index with increased TT prevalence. However, in Sudan we observed the phenomenon of an unexpectedly-positive association between community-level TT prevalence and aridity index. This counter-intuitive relationship may be attributed to coinfections facilitated in humid climates. It has been shown that coinfection with other bacteria [53], such as *Streptococcus pneumoniae* and *Staphylococcus aureus* [54], could influence progression of TT [55].

High levels of self-reported latrine use by adults, aridity index and 1997 night light had strongly significant associations with TT prevalence in only three of ten countries. This suggests that hygiene practices, dry climate and historic infrastructure may link to increased community-level TT prevalence in some settings, but generally they do not. Previous studies have clearly shown the association between access to WASH and risk of TF [25, 32, 39, 56] and so it is not surprising that our models, which account for TF prevalence, generally do not demonstrate significant residual associations between TT and WASH variables. The variation in direction of association may be an artefact of WASH improvements over time, or—hypothetically—existence of latrines themselves could contribute to facilitating *M. sorbens* breeding if the latrines are not appropriately maintained, thereby deterring some potential users whilst protecting householders from legal or peer pressure to build an adequate facility. The development of TT requires many previous *C. trachomatis* infections [4] and so populations that historically had poor WASH access may now have high TT burdens, even if the WASH situation has since improved.

Many other studies have identified correlates of high TF prevalence, including potential socio-economic, demographic and environmental risk factors [25, 32, 39, 56–58], and have explored TF’s spatial distribution at different geographic scales [33, 52, 59–61]. However, few studies have specifically examined TT’s environmental risk factors and spatial distribution [42, 62, 63]. These previous studies were limited by the amount of data they incorporated, and their conclusions therefore had constrained generalizability. Our models, developed using large datasets from ten countries with outcome data considered to be gold standard [64], reached similar conclusions and so provide additional validation to this previous work.

The variation between countries in directions of association of environment-related indicators and the variation in spatial structure indicate that fitting a single model to the whole set of data is inadvisable. These variations are presumably attributable to country context.

There may be several explanations for the inconsistency of associations between large-scale indicators and community-level TT seen between countries. Studies have shown that post-operative recurrence of TT [55, 65, 66] and incidence of scaring [67, 68] may be important influencers of TT prevalence. It would be valuable for future models to further explore these elements. Our modelling approach did not capture recent or historical population movement. Migration could certainly play a role in the geographic distribution of TT. It is also important to note that different ethnic groups may have different progression rates to TT. For example, a study in the Gambia found a polymorphism in the TNF-α gene promoter was associated with scarring, and was found more frequently among Mandinkas than other ethnic groups [69].

We observed residual spatial correlation in only five countries (Benin, DRC, Ethiopia, Mozambique, and Sudan), suggesting that in the remaining countries there are no outstanding large-scale environmental factors influencing progression to TT.

In the geostatistical models, we identified a very rapid decline in spatial correlation with distance at larger scales, after accounting for covariates. This suggests that very closely adjacent communities have similar levels of TT.

## Conclusion

The lack of consistent risk factors beyond community-level TF raises concerns that the models identified artefacts that are not generalizable, such as non-trachomatous trichiasis, or that the clinical history of trachoma varies substantially between settings. This underlines the importance of understanding local context when designing interventions for at-risk populations. Whilst our findings are not generalizable across countries, they can provide general direction for where to initiate case finding activities. As has been found in the Guinea Worm eradication program, active surveillance and case finding will be essential as trachoma elimination endpoints draw closer [70]; these activities become more expensive as prevalence drops [71]. This uniquely large and standardized analysis provides important insight into the variation in community-level TT distribution and identifies substantial variation in the relationship between community-level TF and TT prevalence. For some countries, important environmental risk factors were identified which can be used to inform case finding efforts, by providing insight into where TT cases are most likely to be found. Our findings suggest that in some countries it is possible to inform strategic location of TT management services, potentially improving efficiency of the end-game of trachoma elimination.

## Figures and Tables

**Fig. 3:**
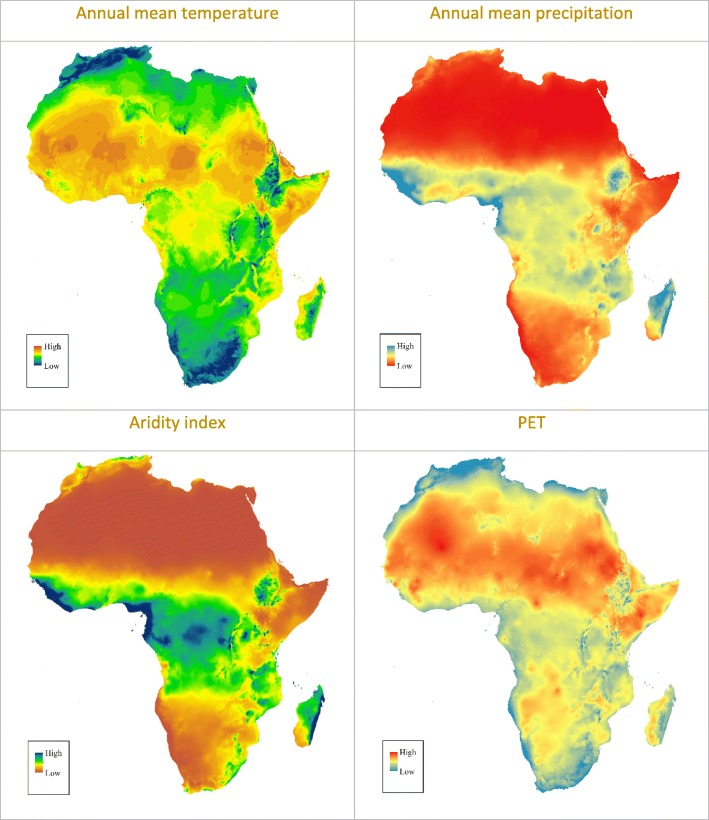
5 km gridded climate raster maps. Data depicted in this map were obtained from the Consortium for Spatial Information (CGIAR-CSI) [41]. The georeferenced raster files were converted to a 5 km × 5 km resolution and visualized by the manuscript authors using ArcGIS 10.1 (ESRI, Redlands, CA, USA)

**Fig. 4:**
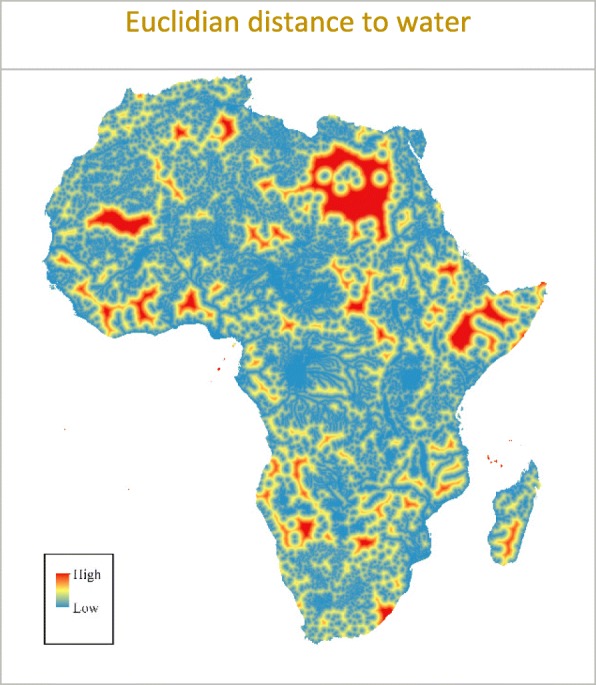
5 km gridded Euclidian distance to water raster map. The manuscript authors produced a continuous surface of distances in km to the nearest water body and permanent rivers based on data obtained from the Global Database of Lakes, Reservoirs and Wetlands [72]

**Fig. 5:**
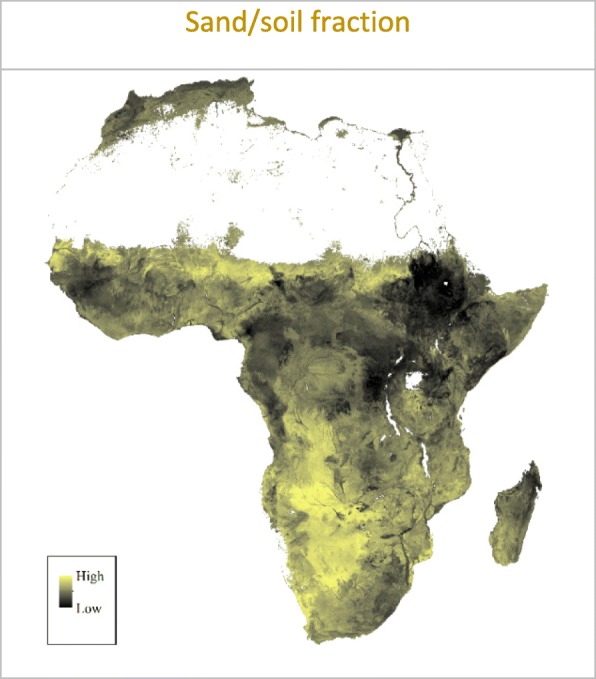
5 km gridded soil composition sand fraction raster map. Data depicted in this map were obtained from the ISRIC-World Soil Information project included in the Harmonized Soil Map of the World [43]. The georeferenced raster files were converted to a 5 km × 5 km resolution and visualized by the manuscript authors using ArcGIS 10.1 (ESRI, Redlands, CA, USA)

**Fig. 6:**
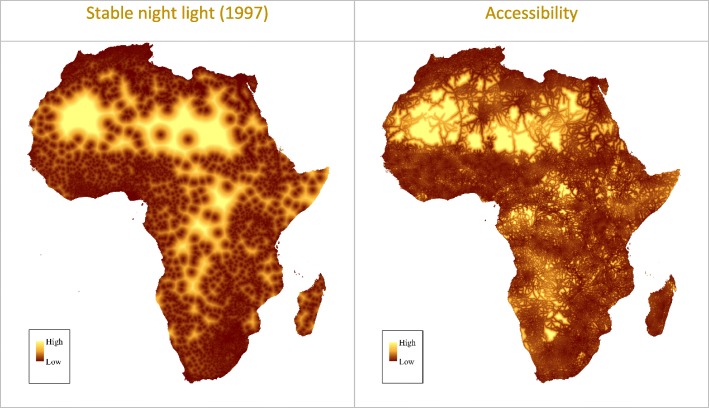
5 km gridded remoteness raster maps. Data depicted in this map were obtained from the Operational Linescan System instrument on board a satellite of the Defence Meteorological Satellite Program [46, 47]. The georeferenced raster files were converted to a 5 km × 5 km resolution and visualized by the manuscript authors using ArcGIS 10.1 (ESRI, Redlands, CA, USA)

## Appendix 1

**Table 7:** GTMP Water, Sanitation, and Hygiene indicators. Responses are classified as improved where marked with X

S1) Where do you and other adults in the household usually defecate? number of households reporting that adults living in the household defecate in either a shared public latrine or a private latrine / number of households enrolled in the survey	1) Shared or public latrine	X
	2) Private latrine	X
	3) No structure, outside near the house	
	4) No structure, in the bush or field	
	9) Other	
S2) Improved latrine: What kind of toilet facility do the adults in the household use? Observed. number of households where an improved latrine is observed / number of households enrolled in the survey	1) Flush/pour flush to piped sewer system	X
	2) Flush/pour flush to septic tank	X
	3) Flush/pour flush to pit latrine	X
	4) Flush/pour flush to open drains	
	5) Flush/pour flush to unknown place	X
	6) Ventilated improved pit latrine (VIP)	X
	7) Pit latrine with slab	X
	8) Pit latrine without slab/open pit	
	9) Composting toilet	X
	10) Bucket	
	11) Hanging toilet/hanging latrine	
	12) No facilities or bush or field	
	99) Other	
H3) Improved Water source: In the dry season, what is the main source of water used by your household for washing faces? number of households reporting to access improved water sources / number of households enrolled in the survey	1) Piped water into dwelling	X
	2) Piped water into yard/pot	X
	3) Public tap/standpipe	X
	4) Tubewell/borehole	X
	5) Protected dug well	X
	6) Unprotected dug well	
	7) Protected spring	X
	8) Unprotected spring	
	9) Rainwater collection	X
	10) Water vendor	
	11) Surface water (e.g. river, dam, lake, canal)	
	99) Other	
H4) Wash near: Washing water: If you collected water there to bring back to the house, how long does it take to go there, get water, and come back? number of households reporting access to improved water sources and those water sources are on the premises / number of households enrolled in the survey	0) All face washing done at the water source	
	1) Water source in the yard	X
	2) Less than 30 min	
	3) Between 30 min and 1 h	
	4) More than 1 h	

## Appendix 2

Climate:

Water access:

Soil composition (fraction and soil properties):

Remoteness:

## Abbreviations

CGIAR-CSI: Consortium for spatial information
DRC: Democratic Republic of the Congo
EU: Evaluation unit
GTMP: Global trachoma mapping project
JMP: Joint monitoring programme for water supply and sanitation
MDA: Mass drug administration
PET: Potential evapo-transpiration
TF: Trachomatous inflammation—follicular
TT: Trachomatous trichiasis
VIF: Variance inflation factor
WASH: Water sanitation and hygiene

## References

[1] Mabey David CW, Solomon Anthony W, Foster Allen (2003). Trachoma. The Lancet.

[2] Thylefors B, Dawson CR, Jones BR, West SK, Taylor HR. A simple system for the assessment of trachoma and its complications. Bull World Health Organ 1987;65(4):477–483. PubMed PMID: 3500800; PubMed Central PMCID: PMC2491032. PMC2491032 3500800

[3] Grayston J. T., Wang S.-p., Yeh L.-j., Kuo C.-c. (1985). Importance of Reinfection in the Pathogenesis of Trachoma. Clinical Infectious Diseases.

[4] Gambhir Manoj, Basáñez Maria-Gloria, Burton Matthew J., Solomon Anthony W., Bailey Robin L., Holland Martin J., Blake Isobel M., Donnelly Christl A., Jabr Ibrahim, Mabey David C., Grassly Nicholas C. (2009). The Development of an Age-Structured Model for Trachoma Transmission Dynamics, Pathogenesis and Control. PLoS Neglected Tropical Diseases.

[5] Burton M, Solomon A. What's new in trichiasis surgery? Community Eye Health 2004;17(52):52–53. PubMed PMID: 17491821; PubMed Central PMCID: PMC1705745. PMC1705745 17491821

[6] Habtamu Esmael, Wondie Tariku, Aweke Sintayehu, Tadesse Zerihun, Zerihun Mulat, Zewudie Zebideru, Kello Amir Bedri, Roberts Chrissy H, Emerson Paul M, Bailey Robin L, Mabey David C W, Rajak Saul N, Callahan Kelly, Weiss Helen A, Burton Matthew J (2016). Posterior lamellar versus bilamellar tarsal rotation surgery for trachomatous trichiasis in Ethiopia: a randomised controlled trial. The Lancet Global Health.

[7] Rajak Saul N., Habtamu Esmael, Weiss Helen A., Bedri Amir, Gebre Teshome, Genet Asrat, Khaw Peng T., Bailey Robin L., Mabey David C.W., Gilbert Clare E., Emerson Paul M., Burton Matthew J. (2012). Epilation for Trachomatous Trichiasis and the Risk of Corneal Opacification. Ophthalmology.

[8] World Health Organization (2017). WHO Alliance for the global elimination of blinding trachoma by the year 2020: progress report on elimination of trachoma, 2017. Wkly Epidemiol Rec.

[9] Migchelsen SJ, Sepulveda N, Martin DL, Cooley G, Gwyn S, Pickering H, et al. Serology reflects a decline in the prevalence of trachoma in two regions of the Gambia. Sci Rep 2017;7(1):15040. doi: 10.1038/s41598-017-15056-7. PubMed PMID: 29118442; PubMed Central PMCID: PMCPMC5678181. 10.1038/s41598-017-15056-7 PMC5678181 29118442

[10] Martin Diana L., Bid Rhiannon, Sandi Frank, Goodhew E. Brook, Massae Patrick A., Lasway Augustin, Philippin Heiko, Makupa William, Molina Sandra, Holland Martin J., Mabey David C. W., Drakeley Chris, Lammie Patrick J., Solomon Anthony W. (2015). Serology for Trachoma Surveillance after Cessation of Mass Drug Administration. PLOS Neglected Tropical Diseases.

[11] Zambrano Andrea I., Sharma Shekhar, Crowley Kathryn, Dize Laura, Muñoz Beatriz E., Mishra Sailesh K., Rotondo Lisa A., Gaydos Charlotte A., West Sheila K. (2016). The World Health Organization Recommendations for Trachoma Surveillance, Experience in Nepal and Added Benefit of Testing for Antibodies to Chlamydia trachomatis pgp3 Protein: NESTS Study. PLOS Neglected Tropical Diseases.

[12] Design and validation of a trachomatous trichiasis-only survey. Geneva: World Health Organization; 2017 (WHO/HTM/NTD/PCT/2017.08). License: CC BY-NC-SA 3.0 IGO.

[13] Evans JR, Solomon AW. Antibiotics for trachoma. Cochrane Database Syst Rev 2011;(3):CD001860. doi: 10.1002/14651858.CD001860.pub3. PubMed PMID: 21412875. 10.1002/14651858.CD001860.pub3 21412875

[14] Dolin PJ, Faal H, Johnson GJ, Minassian D, Sowa S, Day S, Ajewole J, Mohamed AA, Foster A (1997). Reduction of trachoma in a sub-Saharan village in absence of a disease control programme. The Lancet.

[15] Hoechsmann Alex, Metcalfe Nick, Kanjaloti Steve, Godia Henry, Mtambo Olga, Chipeta Towera, Barrows John, Witte Christine, Courtright Paul (2001). Reduction of trachoma in the absence of antibiotic treatment: Evidence from a population-based survey in Malawi. Ophthalmic Epidemiology.

[16] World Health Organization (2016). Validation of elimination of trachoma as a public health problem. WHO/HTM/NTD/2016.

[17] Adamu Mohammed Dantani, Mpyet Caleb, Muhammad Nasiru, Umar Murtala Muhammad, Muazu Habila, Olamiju Francisca, Isiyaku Sunday, Onyebuchi Uwazoeke, Bosso Usman Abubakar, William Adamani, Nwobi Benjamin C., Willis Rebecca, Flueckiger Rebecca Mann, Pavluck Alexandre, Chu Brian K., Olobio Nicholas, Solomon Anthony W. (2016). Prevalence of Trachoma in Niger State, North Central Nigeria: Results of 25 Population-Based Prevalence Surveys Carried Out with the Global Trachoma Mapping Project. Ophthalmic Epidemiology.

[18] Adamu Yilikal, Macleod Colin, Adamu Liknaw, Fikru Wirtu, Kidu Beyene, Abashawl Aida, Dejene Michael, Chu Brian K., Flueckiger Rebecca M., Willis Rebecca, Pavluck Alexandre L., Solomon Anthony W. (2016). Prevalence of Trachoma in Benishangul Gumuz Region, Ethiopia: Results of Seven Population-Based Surveys from the Global Trachoma Mapping Project. Ophthalmic Epidemiology.

[19] Mpyet Caleb, Muhammad Nasiru, Adamu Mohammed Dantani, Muazu Habila, Umar Murtala Muhammad, Abdull Mohammed, Alada Joel, Goyol Musa, Onyebuchi Uwaezuoke, Olamiju Francisca, Isiyaku Sunday, William Adamani, Nwobi Benjamin C., Willis Rebecca, Flueckiger Rebecca Mann, Pavluck Alex, Chu Brian K., Olobio Nicholas, Solomon Anthony W. (2016). Prevalence of Trachoma in Bauchi State, Nigeria: Results of 20 Local Government Area-Level Surveys. Ophthalmic Epidemiology.

[20] Mpyet Caleb, Muhammad Nasiru, Adamu Mohammed Dantani, Muazu Habila, Umar Murtala Muhammad, Alada Joel, Onyebuchi Uwazoeke, Olamiju Fracisca, Isiyaku Sunday, William Adamani, Willis Rebecca, Flueckiger Rebecca Mann, Pavluck Alexandre, Chu Brian K., Mohammed Danjuma, Olobio Nicholas, Solomon Anthony W. (2016). Trachoma Mapping in Gombe State, Nigeria: Results of 11 Local Government Area Surveys. Ophthalmic Epidemiology.

[21] Mpyet Caleb, Muhammad Nasiru, Adamu Mohammed Dantani, Muazu Habila, Umar Murtala Mohammad, Goyol Musa, Onyebuchi Uwazoeke, Chima Ima, Idris Haliru, William Adamani, Isiyaku Sunday, Nwobi Benjamin, Flueckiger Rebecca Mann, Willis Rebecca, Pavluck Alexandre, Chu Brian K., Olobio Nicholas, Solomon Anthony W. (2016). Prevalence of Trachoma in Katsina State, Nigeria: Results of 34 District-Level Surveys. Ophthalmic Epidemiology.

[22] Muhammad Nasiru, Mpyet Caleb, Adamu Mohammed Dantani, William Adamani, Umar Murtala Muhammad, Goyol Musa, Muazu Habila, Onyebuchi Uwaezuoke, Isiyaku Sunday, Flueckiger Rebecca M., Chu Brian K., Willis Rebecca, Pavluck Alexandre L., Alhassan Abdullahi, Olobio Nicholas, Gordon Bruce A., Solomon Anthony W. (2016). Mapping Trachoma in Kaduna State, Nigeria: Results of 23 Local Government Area-Level, Population-Based Prevalence Surveys. Ophthalmic Epidemiology.

[23] Mpyet Caleb, Muhammad Nasiru, Adamu Mohammed Dantani, Muazu Habila, Umar Murtala Muhammad, Goyol Musa, Yahaya Hadi Bala, Onyebuchi Uwazoeke, Ogoshi Chris, Hussaini Tijjani, Isiyaku Sunday, William Adamani, Flueckiger Rebecca M., Chu Brian K., Willis Rebecca, Pavluck Alexandre L., Olobio Nicholas, Phelan Sophie, Macleod Colin, Solomon Anthony W. (2017). Prevalence of Trachoma in Kano State, Nigeria: Results of 44 Local Government Area-Level Surveys. Ophthalmic Epidemiology.

[24] Solomon Anthony W., Pavluck Alexandre L., Courtright Paul, Aboe Agatha, Adamu Liknaw, Alemayehu Wondu, Alemu Menbere, Alexander Neal D. E., Kello Amir Bedri, Bero Berhanu, Brooker Simon J., Chu Brian K., Dejene Michael, Emerson Paul M., Flueckiger Rebecca M., Gadisa Solomon, Gass Katherine, Gebre Teshome, Habtamu Zelalem, Harvey Erik, Haslam Dominic, King Jonathan D., Mesurier Richard Le, Lewallen Susan, Lietman Thomas M., MacArthur Chad, Mariotti Silvio P., Massey Anna, Mathieu Els, Mekasha Addis, Millar Tom, Mpyet Caleb, Muñoz Beatriz E., Ngondi Jeremiah, Ogden Stephanie, Pearce Joseph, Sarah Virginia, Sisay Alemayehu, Smith Jennifer L., Taylor Hugh R., Thomson Jo, West Sheila K., Willis Rebecca, Bush Simon, Haddad Danny, Foster Allen (2015). The Global Trachoma Mapping Project: Methodology of a 34-Country Population-Based Study. Ophthalmic Epidemiology.

[25] Schémann J-F, Sacko D, Malvy D, Momo G, Traore L, Bore O, Coulibaly S, Banou A (2002). Risk factors for trachoma in Mali. International Journal of Epidemiology.

[26] Cumberland Phillippa, Hailu Girum, Todd Jim (2005). Active trachoma in children aged three to nine years in rural communities in Ethiopia: prevalence, indicators and risk factors. Transactions of the Royal Society of Tropical Medicine and Hygiene.

[27] Taylor Hugh R. (1989). Hygiene Factors and Increased Risk of Trachoma in Central Tanzania. Archives of Ophthalmology.

[28] Alene GD, Abebe S. Prevalence of risk factors for trachoma in a rural locality of North-Western Ethiopia. East Afr Med J 2000;77(6):308–312. PubMed PMID: 12858929. 10.4314/eamj.v77i6.46638 12858929

[29] Guraksin A, Gullulu G (1997). Prevalence of trachoma in eastern Turkey. International Journal of Epidemiology.

[30] Roba A. A., Wondimu A., Patel D., Zondervan M. (2010). Effects of intervention with the SAFE strategy on trachoma across Ethiopia. Journal of Epidemiology & Community Health.

[31] West S, Lynch M, Turner V, Munoz B, Rapoza P, Mmbaga BB, et al. Water availability and trachoma. Bull World Health Organ 1989;67(1):71–75. PubMed PMID: 2706728; PubMed Central PMCID: PMC2491213. PMC2491213 2706728

[32] Baggaley R. F., Solomon A. W., Kuper H., Polack S., Massae P. A., Kelly J., Safari S., Alexander N. D. E., Courtright P., Foster A., Mabey D. C. (2006). Distance to water source and altitude in relation to active trachoma in Rombo district, Tanzania. Tropical Medicine and International Health.

[33] Polack S.R., Solomon A.W., Alexander N.D.E., Massae P.A., Safari S., Shao J.F., Foster A., Mabey D.C. (2005). The household distribution of trachoma in a Tanzanian village: an application of GIS to the study of trachoma. Transactions of the Royal Society of Tropical Medicine and Hygiene.

[34] West Sheila K, Emerson Paul M, Mkocha Harran, Mchiwa Wilson, Munoz Beatriz, Bailey Robin, Mabey David (2006). Intensive insecticide spraying for fly control after mass antibiotic treatment for trachoma in a hyperendemic setting: a randomised trial. The Lancet.

[35] Emerson Paul M., Bailey Robin L., Mahdi Olaimatu S., Walraven Gijs E.L., Lindsay Steve W. (2000). Transmission ecology of the fly Musca sorbens, a putative vector of trachoma. Transactions of the Royal Society of Tropical Medicine and Hygiene.

[36] Hafez MAM (1958). Studies on the ecology of Musca sorbens Wied. In Egypt. Bulletin de la Société Entomologiqe d'Egypte.

[37] Courtright P, Sheppard J, Lane S, Sadek A, Schachter J, Dawson C R (1991). Latrine ownership as a protective factor in inflammatory trachoma in Egypt. British Journal of Ophthalmology.

[38] Progress on drinking water, sanitation and hygiene: 2017 update and SDG baseline. Geneva: World Health Organization (WHO) and the United Nations Children’s Fund (UNICEF), 2017 Licence: CC BY-NC-SA 3.0 IGO.

[39] Ramesh Anita, Kovats Sari, Haslam Dominic, Schmidt Elena, Gilbert Clare E. (2013). The Impact of Climatic Risk Factors on the Prevalence, Distribution, and Severity of Acute and Chronic Trachoma. PLoS Neglected Tropical Diseases.

[40] Habtamu Esmael, Wondie Tariku, Aweke Sintayehu, Tadesse Zerihun, Zerihun Mulat, Zewdie Zebideru, Callahan Kelly, Emerson Paul M., Kuper Hannah, Bailey Robin L., Mabey David C. W., Rajak Saul N., Polack Sarah, Weiss Helen A., Burton Matthew J. (2015). Trachoma and Relative Poverty: A Case-Control Study. PLOS Neglected Tropical Diseases.

[41] Zomer RJ, Trabucco A, Bossio DA, Verchot LV (2008). Climate change mitigation: a spatial analysis of global land suitability for clean development mechanism afforestation and reforestation. Agric Ecosyst Environ.

[42] Salim A R, Sheikh H A (1975). Trachoma in the Sudan. An epidemiological study. British Journal of Ophthalmology.

[43] ISRIC. World Soil Information, 2013. Soil property maps of Africa at 1km. www.isric.org.

[44] Henderson Vernon, Storeygard Adam, Weil David N (2011). A Bright Idea for Measuring Economic Growth. American Economic Review.

[45] Chen X., Nordhaus W. D. (2011). Using luminosity data as a proxy for economic statistics. Proceedings of the National Academy of Sciences.

[46] Elvidge CD, Safran J, Tuttle B, Sutton P, Cinzano P, Pettit D (2007). Potential for global mapping of development via a nightsat mission. GeoJournal..

[47] Ebener Steeve, Murray Christopher, Tandon Ajay, Elvidge Christopher C (2005). International Journal of Health Geographics.

[48] Naimi B. R: Uncertainty analysis for SDMs: usdm-package.

[49] Giorgi E, Diggle PJ. PrevMap: an R package for prevalence mapping. J Stat Softw. 2016.

[50] Prost A, Negrel AD. Water, trachoma and conjunctivitis. Bull World Health Organ 1989;67(1):9–18. PubMed PMID: 2650903; PubMed Central PMCID: PMC2491216. PMC2491216 2650903

[51] Hägi Mathieu, Schémann Jean-François, Mauny Frédéric, Momo Germain, Sacko Doulaye, Traoré Lamine, Malvy Denis, Viel Jean-François (2010). Active Trachoma among Children in Mali: Clustering and Environmental Risk Factors. PLoS Neglected Tropical Diseases.

[52] Clements Archie C. A., Kur Lucia W., Gatpan Gideon, Ngondi Jeremiah M., Emerson Paul M., Lado Mounir, Sabasio Anthony, Kolaczinski Jan H. (2010). Targeting Trachoma Control through Risk Mapping: The Example of Southern Sudan. PLoS Neglected Tropical Diseases.

[53] Bobo Linda D., Novak Nicole, Muñoz Beatriz, Hsieh Yu‐Hsiang, Quinn Thomas C., West Sheila (1997). Severe Disease in Children with Trachoma Is Associated with PersistentChlamydia trachomatisInfection. The Journal of Infectious Diseases.

[54] Burton Matthew J., Bowman Richard J. C., Faal Hannah, Aryee Esther A. N., Ikumapayi Usman N., Alexander Neal D. E., Adegbola Richard A., Mabey David C. W., Foster Allen, Johnson Gordon J., Bailey Robin L. (2006). The Long-Term Natural History of Trachomatous Trichiasis in The Gambia. Investigative Opthalmology & Visual Science.

[55] Burton M J (2005). Long term outcome of trichiasis surgery in the Gambia. British Journal of Ophthalmology.

[56] Sahlu T, Larson C. The prevalence and environmental risk factors for moderate and severe trachoma in southern Ethiopia. J Trop Med Hyg 1992;95(1):36–41. PubMed PMID: 1740817. 1740817

[57] Garn Joshua V., Boisson Sophie, Willis Rebecca, Bakhtiari Ana, al-Khatib Tawfik, Amer Khaled, Batcho Wilfrid, Courtright Paul, Dejene Michael, Goepogui Andre, Kalua Khumbo, Kebede Biruck, Macleod Colin K., Madeleine Kouakou IIunga Marie, Mbofana Mariamo Saide Abdala, Mpyet Caleb, Ndjemba Jean, Olobio Nicholas, Pavluck Alexandre L., Sokana Oliver, Southisombath Khamphoua, Taleo Fasihah, Solomon Anthony W., Freeman Matthew C. (2018). Sanitation and water supply coverage thresholds associated with active trachoma: Modeling cross-sectional data from 13 countries. PLOS Neglected Tropical Diseases.

[58] Oswald WE, Stewart AE, Kramer MR, Endeshaw T, Zerihun M, Melak B (2017). Active trachoma and community use of sanitation, Ethiopia. Bull World Health Organ.

[59] KATZ JOANNE, ZEGER SCOTT L, TIELSCH JAMES M (1988). Village and Household Clustering of Xerophthalmia and Trachoma. International Journal of Epidemiology.

[60] Broman Aimee Teo, Shum Kenny, Munoz Beatriz, Duncan Donald D., West Sheila K. (2006). Spatial Clustering of Ocular Chlamydial Infection over Time following Treatment, among Households in a Village in Tanzania. Investigative Opthalmology & Visual Science.

[61] Macharelli Carlos Alberto, Schellini Silvana Artioli, Opromolla Paula Araujo, Dalben Ivete (2013). Spatial distribution of trachoma cases in the City of Bauru, State of São Paulo, Brazil, detected in 2006: defining key areas for improvement of health resources. Revista da Sociedade Brasileira de Medicina Tropical.

[62] Schémann Jean-François, Laffly Dominique, Sacko Doulaye, Zephak Germain, Malvy Denis (2007). Trichiasis and geoclimatic factors in Mali. Transactions of the Royal Society of Tropical Medicine and Hygiene.

[63] Smith Jennifer L., Sivasubramaniam Selvaraj, Rabiu Mansur M., Kyari Fatima, Solomon Anthony W., Gilbert Clare (2015). Multilevel Analysis of Trachomatous Trichiasis and Corneal Opacity in Nigeria: The Role of Environmental and Climatic Risk Factors on the Distribution of Disease. PLOS Neglected Tropical Diseases.

[64] Engels Dirk (2016). The Global Trachoma Mapping Project: A Catalyst for Progress Against Neglected Tropical Diseases. Ophthalmic Epidemiology.

[65] Rajak Saul N., Collin J. Richard O., Burton Matthew J. (2012). Trachomatous Trichiasis and its Management in Endemic Countries. Survey of Ophthalmology.

[66] Rajak Saul N., Makalo Pateh, Sillah Ansumana, Holland Martin J., Mabey David C. W., Bailey Robin L., Burton Matthew J. (2010). Trichiasis Surgery in The Gambia: A 4-Year Prospective Study. Investigative Opthalmology & Visual Science.

[67] West Sheila K., Muñoz Beatriz, Mkocha Harran, Hsieh Yu-Hsiang, Lynch Matthew C. (2001). Progression of active trachoma to scarring in a cohort of Tanzanian children. Ophthalmic Epidemiology.

[68] Burton Matthew J., Rajak Saul N., Hu Victor H., Ramadhani Athumani, Habtamu Esmael, Massae Patrick, Tadesse Zerihun, Callahan Kelly, Emerson Paul M., Khaw Peng T., Jeffries David, Mabey David C. W., Bailey Robin L., Weiss Helen A., Holland Martin J. (2015). Pathogenesis of Progressive Scarring Trachoma in Ethiopia and Tanzania and Its Implications for Disease Control: Two Cohort Studies. PLOS Neglected Tropical Diseases.

[69] Conway DJ, Holland MJ, Bailey RL, Campbell AE, Mahdi OS, Jennings R, et al. Scarring trachoma is associated with polymorphism in the tumor necrosis factor alpha (TNF-alpha) gene promoter and with elevated TNF-alpha levels in tear fluid. Infect Immun 1997;65(3):1003–1006. PubMed PMID: 9038309; PubMed Central PMCID: PMC175081. 10.1128/iai.65.3.1003-1006.1997 PMC175081 9038309

[70] Beyene HB, Bekele A, Shifara A, Ebstie YA, Desalegn Z, Kebede Z, et al. Elimination of Guinea worm disease in Ethiopia; current status of the Disease's, eradication strategies and challenges to the end game. Ethiop Med J 2017;55(Suppl 1):15–31. PubMed PMID: 28878428; PubMed Central PMCID: PMCPMC5582630. PMC5582630 28878428

[71] Fitzpatrick Christopher, Sankara Dieudonné P., Agua Junerlyn Farah, Jonnalagedda Lakshmi, Rumi Filippo, Weiss Adam, Braden Matthew, Ruiz-Tiben Ernesto, Kruse Nicole, Braband Kate, Biswas Gautam (2017). The cost-effectiveness of an eradication programme in the end game: Evidence from guinea worm disease. PLOS Neglected Tropical Diseases.

[72] Lehner Bernhard, Döll Petra (2004). Development and validation of a global database of lakes, reservoirs and wetlands. Journal of Hydrology.

